# Toward Photoactive Wallpapers Based on ZnO‐Cellulose Nanocomposites

**DOI:** 10.1002/gch2.202300034

**Published:** 2023-08-25

**Authors:** Naveed Ul Hassan Alvi, Neha Sepat, Samim Sardar, Magnus Berggren, Isak Engquist, Xavier Crispin

**Affiliations:** ^1^ RISE Research Institutes of Sweden Södra Grytsgatan 4, plan 2 Norrköping 60233 Sweden; ^2^ Laboratory of Organic Electronics ITN Linköping University Norrköping SE‐60174 Sweden; ^3^ Center for Nano Science and Technology@PoliMi Istituto Italiano di Tecnologia (IIT) Via Giovanni Pascoli 70/3 Milano 20133 Italy; ^4^ Wallenberg Wood Science Center ITN Linköping University SE‐601 74 Norrköping 60174 Sweden

**Keywords:** anti‐microorganism wallpapers, cellulose pulp fiber, large‐scale fabrication, photocatalytic and photoconductive, ZnO nanowires

## Abstract

The quest for eco‐friendly materials with anticipated positive impact for sustainability is crucial to achieve the UN sustainable development goals. Classical strategies of composite materials can be applied on novel nanomaterials and green materials. Besides the actual technology and applications also processing and manufacturing methods should be further advanced to make entire technology concepts sustainable. Here, they show an efficient way to combine two low‐cost materials, cellulose and zinc oxide (ZnO), to achieve novel functional and “green” materials via paper‐making processes. While cellulose is the most abundant and cost‐effective organic material extractable from nature. ZnO is cheap and known of its photocatalytic, antibacterial, and UV absorption properties. ZnO nanowires are grown directly onto cellulose fibers in water solutions and then dewatered in a process mimicking existing steps of large‐scale papermaking technology. The ZnO NW paper exhibits excellent photo‐conducting properties under simulated sunlight with good ON/OFF switching and long‐term stability (90 minutes). It also acts as an efficient photocatalyst for hydrogen peroxide (H_2_O_2_) generation (5.7 × 10^−9^ m s^−1^) with an envision the possibility of using it in buildings to enable large surfaces to spontaneously produce H_2_O_2_ at its outer surface. Such technology promise for fast degradation of microorganisms to suppress the spreading of diseases.

## Graphical abstract

The quest for eco‐friendly materials with anticipated impact on sustainability is one of the prime human duties of this century. For this task, several classical strategies of composite materials can be applied on nanomaterials for an array of sustainability applications. Besides the actual technology and applications, themselves, also processing and manufacturing methods should be further advanced to make entire technology concepts sustainable. Here, an efficient way is shown to combine two low‐cost materials, cellulose and zinc oxide (ZnO), to achieve novel functional and “green” materials via paper‐making processes.

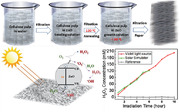

## 1 Introduction

Cellulose isone of the main components in plants (≈60–70% of their solid content). It is a remarkable biopolymer with excellent chemical and mechanical stability. It is renewable at large scales, easily recyclable, non‐toxic, and biodegradable; thus, it is a key material for sustainability and green applications. These days, the highest cellulose consumption is for papermaking, textile, and non‐woven applications (e.g., filters); recent research foresees many other exciting possibilities, such as in printed electronics, where paper is the substrate onto which new (opto)electronic devices can be printed.^[^ ^1^ ^]^ A new trend is the exploration of composites combining cellulose fibers with organic/inorganic materials. These innovations are leading to many new applications for cellulose as a green carrier of electronics and photonics, including chemical/biosensors, UV and strain sensors, solar cells, light‐emitting diodes, photoelectrodes, piezoelectric energy harvesters, and batteries.^[^ ^2^, ^3^, ^4^, ^5^ ^]^

Among the interesting materials that can be combined with cellulose, we will here focus on ZnO. It is an attractive metal oxide owing to its remarkable high photoreactivity with chemical stability, high earth‐abundance, and low environmental toxicity.^[^ ^6^, ^7^, ^8^, ^9^, ^10^ ^]^ It has a direct wide bandgap of 3.4 eV with large exciton binding energy (60 m eV) and can with high electron mobilities that can reach as high as 1000 cm^2^ V^−1^ s^−1^ for a single nanowire.^[^ ^11^, ^12^ ^]^ ZnO can be fabricated in a large variety of nanostructures: nanoparticles (NPs, nanowires (NWs), nanotubes, nanowalls, tetrapods, nanoflowers, nanobelts, etc.) using low temperature (<100 °C), facile, cost‐effective, and scalable fabrication techniques.^[^ ^13^, ^14^ ^]^ Due to the several basic properties of ZnO, micro‐ and nanoscale ZnO material systems are commonly included in sunscreens, sunglasses, coatings, and paints to provide strong protection against UV radiation. ZnO also plays a vital role in various industries such as rubber, electronics and photonics, pharmaceuticals, and food.^[^ ^15^, ^16^ ^]^

ZnO has been incorporated as an antimicrobial compound into cotton fabrics, surface coatings, cosmetics, and food packaging. It is one of the most widely used nanomaterials as an antibacterial agent. Direct contact of ZnO nanoparticles with the cell membrane also produces rupture of the bacterial cell wall due to the presence of surface defects which cause eventually cell death.^[^ ^15^, ^17^, ^18^ ^]^ While ZnO nanoparticles are dangerous for bacteria, they show some level of toxicity for fishes (LD50 is 10 mg kg^−1^)^[^ ^19^, ^20^ ^]^ and a low level of toxicity in oral intake for the human body (2000 mg kg^−1^ for NPs). More interestingly in the context of this study, ZnO has been demonstrated as the key ingredient for the photo‐induced removal of organic compounds from contaminated air and water.^[^ ^15^, ^16^, ^17^, ^21^ ^]^ Different mechanisms for disinfection have been reported for the antibacterial activity of ZnO upon light irradiation. By absorbing UV light, the photocatalytic activity of ZnO produces reactive oxygen species that includes hydroxyl radicals (^•^OH), superoxide ions (O^−^_2_), and hydrogen peroxide (H_2_O_2_), which are known to cause oxidative stress and phototoxic effects on bacteria.^[^ ^22^ ^]^ Nanoscale materials provide enhanced properties as compared to microscale due to that their larger surface area improves surface reaction rates.

Suspensions of nanoparticles in electrolytes have been used to terminate bacteria^[^ ^23^ ^]^ with the drawback that a later separation is needed to remove the nanoparticles from the water treatment system. The release of nanoparticles into aquatic systems may pose a threat to non‐targeted organisms such as fish, crustaceans, and algae. The immobilization of ZnO nanostructures on solid substrates would be highly advantageous. There are various reports on how to grow different ZnO nanostructures on various flexible plastics, paper, and textile substrates using comprehensive growth strategies.^[^ ^24^, ^25^, ^26^, ^27^, ^28^ ^]^ Cotton fibers were decorated with organic/inorganic nanostructures.^[^ ^29^ ^]^ ZnO nanowires have been integrated onto the surface of cotton fibers by hydrothermal synthesis.^[^ ^30^ ^]^ Cellulose nanocrystals were decorated with titanium dioxide (TiO_2_) nanorods and Au (gold) crystals.^[^ ^31^ ^]^ The addition of ZnO tetrapod crystals (TPCs) or nanoparticle powders with cellulose fibers led to a semiconducting paper with several opportunities for applications in electrochemical devices and optoelectronic systems.^[^ ^32^, ^33^ ^]^ One of the major challenges is to avoid mechanical detachment of the ZnO micro‐and nanoparticles from the paper over time.

In this work, we have grown highly dense ZnO NWs forest on cellulose‐pulp‐fibers through a simple aqueous‐chemical approach applied before the papermaking process. This strategy to synthesize functional cellulose fibers enables the introduction of photo‐conductive and catalytic functions inside the paper. Imortantly those functional cellulose fibers are fully compatible with large‐scale paper manufacturing process, thus openeing a realistic route to produce semiconductor papers The chemical growth of ZnO NWs solves the issue of losses of small‐sized ZnO particles during the dewatering process. Such an issue was found with a simple  mix of cellulose fibers and ZnO particles. The strategy to grow ZnO NWs forest on the fibers leads to higher photoconductivity compared to other strategies integrating ZnO NPs and ZnO TPCs into paper. The resulting photocatalytic property of the semiconducting paper was explored by measuring the rate of production of H_2_O_2_ achieved under sunlight exposure. This work paves the way for scalable semiconducting papers for photoconductive and photocatalytic applications such as low‐cost and large‐volume water purification systems or antimicrobial wallpapers.

## 2 Results and Discussion

**Figure 1:**
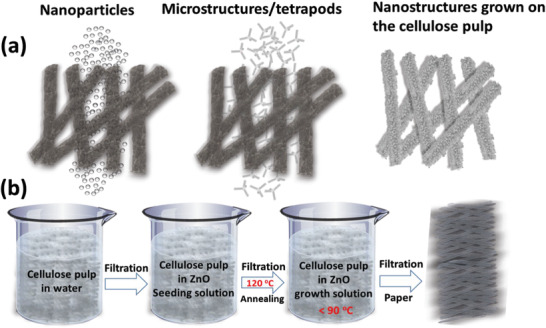
a) Schematic illustration of the type of the problem solved, ZnO NPs (left) and ZnO TPCs (middle) don't attach to the cellulose fibers but pass through the paper. On the contrary, the ZnO NWs (right) grown on the cellulose pulp are well attached and don't escape upon dewatering. b) Schematic illustration of main steps for the growth of ZnO NWs on cellulose pulp.

**Figure 2:**
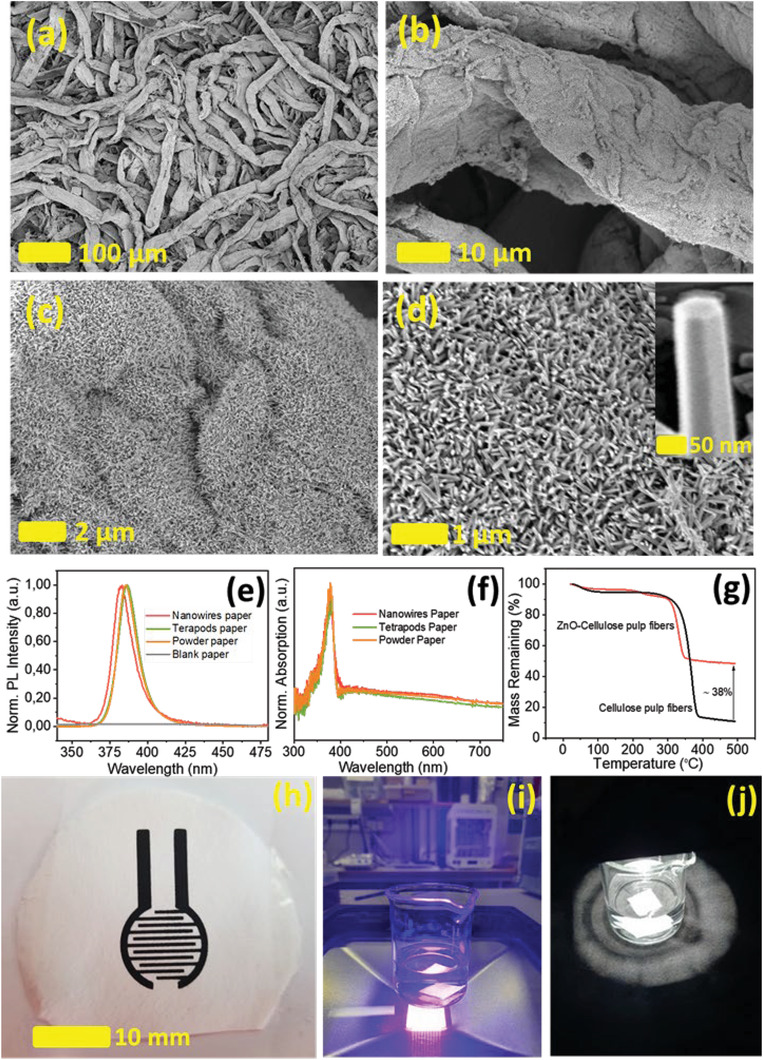
a–d) SEM images of ZnO NWs grown on the surface of cellulose pulp fibers. e) Normalized absorption spectra of the paper layer made of cellulose and, respectively, ZnO NWs, ZnO TPCs, and ZnO NPs paper. Measurement taken at room temperature. f) Normalized photoluminescence spectra of the ZnO NWs, ZnO TPCs, ZnO NPs, and blank paper (not normalized) at room temperature upon excitation at 355 nm. g) Thermogravimetry patterns of ZnO NWs/cellulose‐pulp‐fiber and cellulose‐pulp‐fibers. h) Photograph of fabricated ZnO NWs paper with printed electrodes. i,j) Photographs of the photochemical experiment with ZnO NWs paper in water, irradiated with a violet source and simulated sunlight.

The process protocol of deriving pulp fibers from wood tissue is normally achieved through different mechanical and chemical manufacturing steps. The fabricated semiconducting papers are produced from pulp fibers with the following inclusion of various ZnO particle material systems. Three types of cellulose‐ZnO composites were fabricated, see **Figure** **1**a. The first semiconducting paper is obtained by mixing 0.45 g of ZnO nano‐powder (NP) with 1 g of cellulose fibers dispersed in water. Since the intention is to find an appropriate scalable manufacturing method to make semiconducting papers, our goal is to retain as much ZnO particle as possible during the dewatering step of paper production. The ZnO NPs + cellulose water dispersion was dewatered by using a wire mesh acting as the filter with a pore size of 10 µm. About 0.09 g of ZnO NPs passed through the wire during dewatering, which results in a nanocomposite with a dry weight ratio of 0.36 g of NPs powder per 1 g of cellulose fibers. The second semiconducting paper was fabricated by mixing 0.45 g ZnO TPCs with 1 g of cellulose fibers and in this case, 0.16 g of the ZnO TPC content leaked out during the filtration process. The resulting composition of the second paper was 0.29 g of TPC powder per 1 gram of cellulose fibers. We thus found the loss of ZnO NPs during the dewatering step is a major issue for scaling up the fabrication of ZnO paper with both both nanparticles (ZnO NPs) and microparticles (ZnO TPCs).Thus, we proposed a third strategy with the target of considerably improving the retention of the ZnO content in the paper. Cellulose fibers, precoated with a seed layer, were introduced in a water solution comprising a ZnO precursor, which resulted in a chemical hydrothermal growth of ZnO nanowires (NWs) directly on the fiber surface. The resulting cellulose fiber system, functionalized with ZnO NWs, was filtered using the wire mesh to remove water. After analysis, no NWs were found in the filtrate, which indicates that the ZnO NWs attach strongly to the cellulose fibers (Figure 1b). Thermogravimetric investigations revealed that the resulting weight ratio of the two components after this synthesis protocol is 0.38 g of ZnO NWs per gram of cellulose fibers, see **Figure** **2**g.

The investigations were then focused on the cellulose pulp fibers functionalized with ZnO NWs produced through the low‐temperature aqueous chemical growth process and compared such ZnO NW paper to the other ZnO papers. The scanning electron microscopy (SEM) images indicate that a uniform and dense ZnO NWs forest is grown perpendicular to the surface of the cellulose fibers. The average diameter of the fabricated ZnO NWs is ≈70 nm and the estimated average length reaches ≈300–400 nm, see Figure 2a–d. Figure 2e displays the optical absorption spectra of the ZnO NWs, ZnO TPCs, and ZnO NPs papers are measured at room temperature.  A strong absorption band attributed to ZnO is observed at 378 nm in the UV domain, confirming that the grown nanowires truly are based on ZnO chemical structure. Figure 2f gathers the normalized photoluminescence spectra of the three papers (ZnO NWs, ZnO TPCs‐ and ZnO NPs) and the blank paper. Those measurements are performed at room temperature upon excitation at 355 nm. The emission spectra peak at 382 nm for ZnO NWs and at 386 nm for ZnO TPCs and ZnO NPs. THese are typical wavelength values for the band edge emission in ZnO crystals of goodquality.^[^ ^34^ ^]^

**Figure 3:**
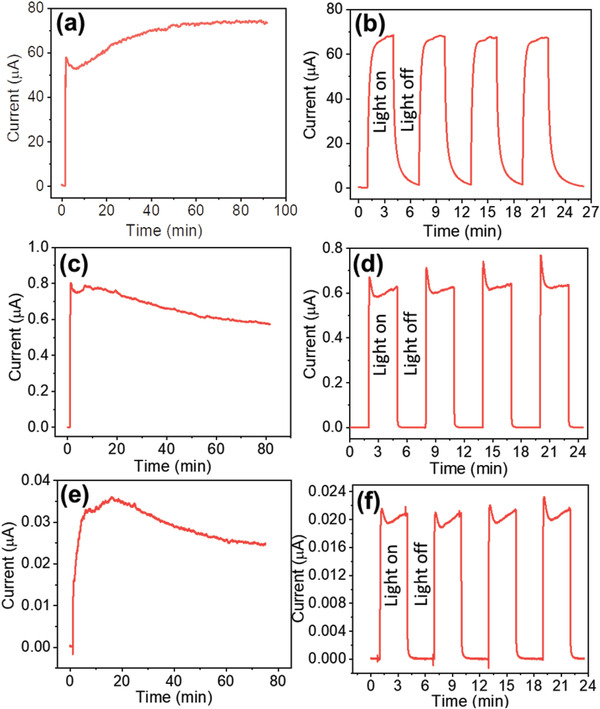
a,b) Photo‐response and ON/OFF switching performance of the ZnO‐NWs paper device under an irradiance of 1 Sun at 1 V, similarly (c,d) and (e,f) are shown for the ZnO‐TPCs and ZnO‐NPs paper devices, respectively.

The photoconductivity of semiconducting papers (ZnO NWs, ZnO TPCs, and ZnO NPs) with two interdigitated electrodes was measured under the irradiance of 1 Sun (1000 W m^−2^) for a period of 75–90 min at a constant biasing of 1 V, see **Figures** **3**a,c,e. The measured photocurrents of the semiconducting papers were around 68 µA with ZnO NWs, 0.6 µA with ZnO TPCs, and 0.02 µA with ZnO NPs (estimated from Figure 3b,d,f. The ZnO NWs‐based paper showed ≈100 times higher photocurrent as compared to ZnO TPCs‐based paper and 3000 times higher photocurrent compared to the ZnO NPs‐based paper. This is remarkable since the weight content of ZnO NWs in the resulting paper is 10 times lower than the other ZnO paper with TPCs and NPs. This likely reflects the good interconnection between the grown nanowires formed on the cellulose fiber being arranged as an interconnected forest. Also, the photocurrent for the ZnO NWs‐based paper gradually increased and stabilized over a 90‐min period, while the other papers showed a photocurrent level that gradually decreased over similar time periods (Figure 3a,c,e). The ZnO NWs‐based paper does not only have good interconnectivity from an electrical point of view, but its performance is also stable. This interconnectivity is maybe counterintuitive because the NWs stand perpendicular to the surface and not all of them are in contact with each other, except along the basal surface (see, e.g., Figure 2d). The hypothesis is that the fraction of the ZnO NW coating closest to the fiber surface is relatively more interconnected as compared to the outermost content.

The ON/OFF switching performance of the three semiconducting papers was measured under 1 Sun intensity by applying light pulses (light/dark), each lasting for 3 minutes. Photocurrent pulses for all devices show good stability and reversibility with a rapid rise/fall under continuous light/dark pulsing (Figures 3b,d,f). The photoconductive sensitivity can be calculated as:

(1) $$\mathrm{Photoconductive}\;\mathrm{sensitivity}=\frac{I_{\max}-I_{\min}}{I_{\min}}$$

The ZnO NWs‐based paper was found to have a sensitivity of around 80, where *I*_max_ = 68.40 µA and *I*_min_ = 0.847 µA. The residual conductivity in the dark may correspond to the formation of the electron's depletion region near the surface of the ZnO NWs.^[^ ^35^, ^36^ ^]^

**Figure 4:**
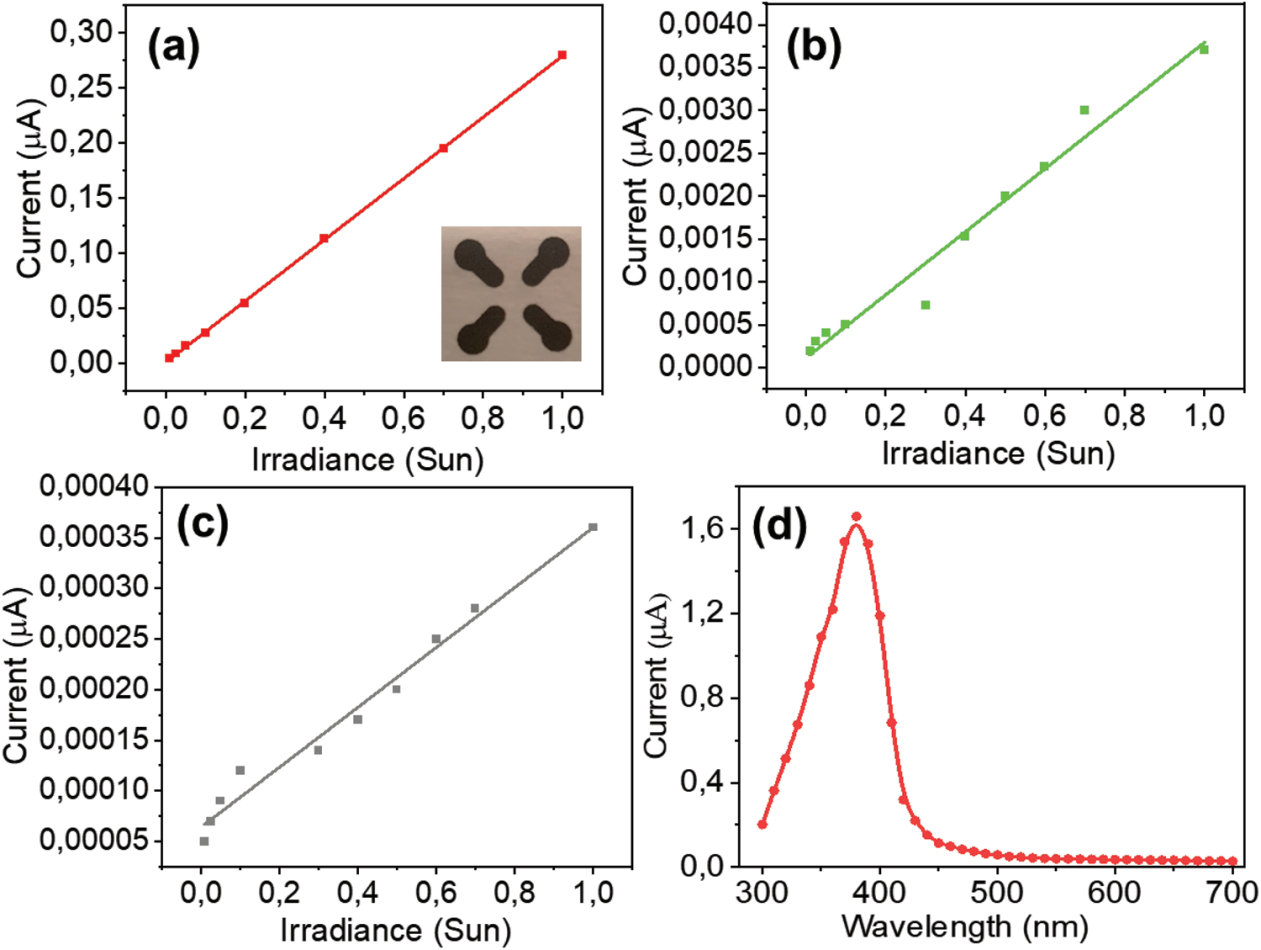
Panel (a) shows photocurrent versus irradiances (0.01–1 Sun) at 1 V for the ZnO NWs‐based paper with a four‐probe device. The data line is a linear fit of the average photocurrents. Similarly, panel (b) shows for the ZnO‐TPCs paper device and panel (c) shows for the ZnO‐NPs paper device. Panel (d) shows the UV photocurrent of the ZnO‐NWs paper device at 1 V versus the irradiation wavelength.

The photocurrents were measured as a function of irradiance (0.01–1.0 Sun) using four‐probe electrodes (**Figure** **4**). Using this setup, it could avoid shadowing effects caused by the probes/electrodes could be avoided as they may otherwise influence the photoconductivity of the paper. (Note that two‐probe measurement confirms the conclusion (see Supplementary Information). A fixed voltage (1 V) was applied between two opposite probes, and the photocurrent was measured between the other two opposite probes at the same time as the light intensity was increased stepwise from 0.01 to 1.0 Sun. Figure 4a shows the photocurrent measured for NWs paper devices while Figure 4b,c display the photocurrent for ZnO‐TPCs and ZnO NPs‐based paper devices, respectively.

From the fixed applied voltage (1 V) and measured photocurrent (*I*) at an irradiance of 1 Sun, the sheet resistance (*R*_sheet_) at 1 Sun was estimated using,^[^ ^31^ ^]^

(2) $$R_{\mathrm{sheet}}=\frac{\pi }{\ln2}\;\frac{V}{I}$$

The *R*_sheet_ of the ZnO NW‐paper device was around 16 MΩ sq^−1^ at 1 Sun illumination while the ZnO TPCs and ZnO NPs‐based papers showed significantly higher values of ≈1200 and 12 600 MΩ sq^−1^.

The spectral sensitivity of the ZnO NWs paper was also investigated by measuring the photo‐current versus wavelengths in the range of 300–700 nm. Figure 4d displays the spectral photo‐power (fixed biased of 1 V) versus wavelengths, which as expected resembles the absorption spectra of the ZnO NWs paper (Figure 2e).

**Figure 5:**
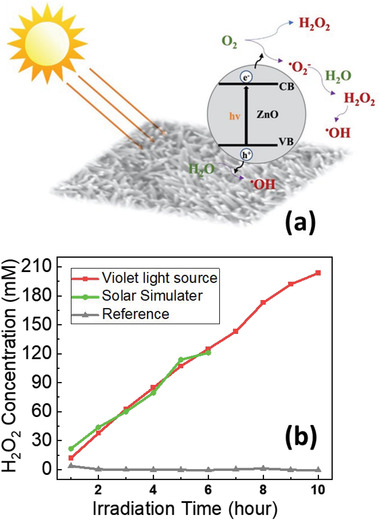
a) Schematic of the photochemical reaction mechanism of ROS generation under light irradiation. b) Time‐dependent photochemical evolution of peroxide by ZnO NW paper irradiated with a violet source and simulated AM1.5G sunlight irradiation (2.25 cm^2^ size sample immersed in 20 mL water). The reference is the cellulose paper without ZnO NWs.

The 4‐order of magnitude higher photocurrent per ZnO weight mass of the ZnO NWs‐paper, as compared to the two other ZnO‐based papers, motivated us to investigate its photocatalytic properties. Photocatalytic reactions were performed by suspending the ZnO NWs‐based paper in pure H_2_O under simulated sunlight or violet light irradiation without any electron donor agents added to the liquid and without oxygen purging. To achieve efficient photosynthesis, the irradiation wavelength of the light source should match the bandgap of the photocatalyst. From the optical absorption spectrum (Figure 2e) and photocurrent spectra measurements (Figure 4d), it is found that the ZnO NW paper has strong absorption in the UV domain because of its bandgap (3.2 eV). The photo‐excitons can be separated into electrons and holes upon irradiation with light beyond the optical bandgap energies of the photocatalyst material. The electrons will be excited to the conduction band leaving holes in the valance band (**Figure** **5**a).

Let us investigate the fate of the photogenerated electrons. They are migrating within the ZnO NW structures to diffuse to its surface in contact with the aqueous medium including dissolved dioxygen. The electrons are involved in the reduction of dioxygen to produce reactive oxygen species depending on their protonation levels: superoxide ions O_2_^●–^, hydroperoxyl HO_2_^●^ and H_2_O_2_.^[^ ^14^, ^17^ ^]^ Figure 5b shows the H_2_O_2_ generation rate on a piece of ZnO‐NWs paper (≈70 mm^3^ in volume). The production leads to 121 µm of H_2_O_2_ in the water within 6 h under the solar simulator (AM1.5 = 1 kW m^−2^). This gives a calculated rate of H_2_O_2_ production of about 5.7×10^−9^ m s^−1^. An almost constant rate was measured during a period of 6 h, which indicates stable photocatalytic activity. With the violet light source (λ_max_ = 400 nm, 1.2 kW m^−2^), the concentration of peroxide reached 203 µm for a total period of ten hours of irradiation. The rate of peroxide production (calculated from the slope of Figure 5b) was approximately constant (5.9×10^−9^ m s^−1^). Almost the same rate of peroxide production was achieved regardless of the light source, indicating that photons are not the limiting factor for the reaction. Oxygen can be viewed as the primary limiting factor. To further boost the production of reactive oxygen species, more oxygen can be injected into water, or electron donors (hole acceptors) can be added to the solution.

Negligible H_2_O_2_ was generated when bare cellulose paper was exposed to light from a solar simulator (as well as with violet light), which confirms that the photocatalytic reaction requires the ZnO NWs for the formation of H_2_O_2_ (reference in Figure 5b). It was also experimented with the photocatalytic papers without irradiation (i.e., in dark conditions); then no H_2_O_2_ was detected. This confirms that H_2_O_2_ evolution relies on the photocatalytic activity of the ZnO NWs. For water cleaning purposes, it is the chemical interaction of hydrogen peroxide with bacteria that determines the integrity of bacterial cell walls, DNA, and cellular proteins.^[^ ^17^, ^19^, ^21^, ^22^ ^]^ Note that the oxygen reduction reaction in a neutral environment leads to the production of hydrogen peroxide through the following reduction reaction O_2_ + H_2_O + 2e^−^ → HO_2_^−^ + OH^−^ with the acid equilibrium HO_2_^−^ + H^+^ → H_2_O_2_. The neutral environment turns rapidly to vary basic pH upon this electrochemical reaction. Both the basic pH and the H_2_O_2_ are known to degrade cellulose.^[^ ^37^ ^]^

Regarding the fate of the photogenerated holes, it is believed that the main path is an oxidation of the cellulose in the close vicinity of ZnO NWs. The electrochemical oxidation of cellulose in the basic medium is known to lead to the formation of carbonyl groups on the cellulose.^[^ ^38^ ^]^ This oxidation of cellulose with the photogenerated holes in ZnO NW occurs at the same time as the reduction of oxygen into H_2_O_2_. This explains the high H_2_O_2_ production rate obtained in pure water with functionalized cellulose without supplementing any additional sacrificial agent (electron donor).^[^ ^39^ ^]^ The eventual drawback of the approach of using sacrificial cellulose is that the paper will eventually degrade with time. However, this effect was not studied and seen during the time scale of the experiment.

These strategies can be pursued if the goal is to produce photo‐catalytically H_2_O_2_ as a green chemical fuel and industrial oxidant. In that vein, large‐scale semiconducting paper is also an attractive route for a new sustainable photochemical industry.

## 3 Conclusion

In summary, ZnO NWs are integrated on cellulose pulp fibers to be able to produce semiconducting paper with a papermaking process without losses of ZnO particles upon dewatering. A low‐cost and straightforward aqueous chemical process is adopted that doesn't need a cleanroom environment to fabricate the semiconducting paper. The fabricated ZnO NWs‐based cellulose paper is highly photoconductive under simulated sunlight. When the ZnO NW paper is dipped into liquid water and exposed to solar light, dissolved oxygen from the air is photoreduced to produce H_2_O_2_ at a constant rate for up to ten hours.

As an exciting perspective, the scalability of the manufacturing process compatible with papermaking envision a true feasibility for semiconducting ZnO‐paper. This could have an impact on water purification under sunlight. The hygroscopicity of cellulose (ability to absorb water) and the photocatalytic activity of ZnO make this semiconducting paper a potential surface that spontaneously produces H_2_O_2_ as an antimicrobial and antibacterial agent. As the coronavirus disease 2019 (COVID‐19) has been demonstrated to be sensitive to H_2_O_2_, this finding opens a perspective for making wallpapers in buildings that destroy microorganism contamination and reduces the spread of diseases.

## 4 Experimental Section

### Synthesis of ZnO NWs on Cellulose Pulp Fibers

The ZnO NWs are grown on the cellulose‐pulp‐fibers at a low temperature (<100 °C) by using a facile and cost‐effective aqueous chemical growth method described by Vayssieres et al.^[^ ^40^ ^]^ This method was modified with the application of a ZnO seeding solution technique introduced by Womelsdof et al.^[^ ^29^ ^]^ Using this method, 0.01 m zinc acetate dihydrate (99.9% purity, Sigma–Aldrich) was mixed in methanol. This solution was heated to 60 °C for 2 h under continuous stirring. During this process, 0.03 m potassium hydroxide (90% purity, Sigma–Aldrich) was mixed in methanol dropwise. The water was removed from the cellulose pulp by filtration. A thin and uniform seeding layer of ZnO was formed by dipping the pulp cellulose in the ZnO seeding solution for 3 min. The extra solution was removed by filtration. An equimolar concentration (0.1–0.75 m) of zinc nitrate hexahydrate (Zn(NO_3_)_2,_ 6H_2_O) (99% purity, Sigma–Aldrich) and hexamethylene‐tetramine (HMT, C_6_H_12_N_4_) (99% purity, Sigma–Aldrich) were mixed to prepare the growth solution. The cellulose pulp was mixed in the solution and the solution container was placed in a furnace at 90 °C for 3–5 h.

The growth of ZnO NWs proceeded through the following reactions.^[^ ^30^ ^]^

In the first step, the reaction between HMT (C_6_H_12_N_4_) and water produced ammonia,

(3) $${\left({\mathrm{CH}}_{2}\right)}_{6}N_{4}+6H_{2}O\to 6\mathrm{HCHO}+4NH_{3}$$

In the second step, the reaction between ammonia and water splitted ammonia into ammonium and hydroxide ions,

(4) $${\mathrm{NH}}_{3}+H_{2}O\to {\mathrm{NH}}_{4}^{+}+OH^{-}$$

In the third step, the reaction between hydroxide ions and zinc ions ended up with the growth of solid ZnO NWs,

(5) $$2OH^{-}+Zn^{+2}\to {\mathrm{ZnO}}_{\left(s\right)}+H_{2}O$$

After the growth, the ZnO NWs were found on the surface of the cellulose fibers. The growth of ZnO NWs on cellulose‐pulp‐fibers was performed several times and the results were reproducible.

### Paper Manufacturing

ZnO TPCs with an average size of 20 µm (single crystal with purity 99.9%) were purchased from AMTEC KK, Osaka, Japan. The ZnO NPs with a particle size of <1 micron (single crystal, 99.9% purity) was purchased from Sigma–Aldrich. The functional papers (with either tetrapods or nanoparticles) were made by mixing the cellulose fibers in water with the ZnO particles (45 wt.% solid content in the composite). This was followed by a filtering step.

The mixture of water and ZnO NWs grown on pulp fibers was filtered by using a simple filtration process. For the three types of ZnO papers, the filter pore size was ≈10 microns. The water was filtered out and a uniform layer of ZnO‐cellulose pulp fibers was formed on the filter. The size of the fabricated paper was ≈2 inches in diameter. It was dried at 100 °C for 1 h and was pressed under a weight of one ton.

### Electrode Printing

The carbon electrodes were printed on the fabricated ZnO NWs paper by using a specially designed screen‐printing mask and commercially purchased carbon ink. Two‐probe interdigitated electrodes as shown in Figure 2g with electrode width of 0.5 mm, distance between electrodes of 1 mm, and total area of 1.13 cm^2^ were printed to measure the photo‐sensing properties while four‐probe square‐designed electrodes as shown in the inset of Figure 4a with electrode width 2 mm and separation between opposite electrodes 3 mm and total area between the electrodes was 9 mm^2^ were printed to measure the sheet resistance (Rs). After printing, the devices were kept in a furnace at 120 °C for 5 min under normal ambient condition.

### Measurements

Morphological and structural characterizations of ZnO NWs, TPCs, and NPs were conducted by using a scanning‐electron‐microscope (SEM, Sigma 500 Gemini).

Absorption measurements were performed at room temperature using a 300 W xenon lamp as the light source (Newport). The reflected light from the sample was captured using a light‐guide and a spectrograph (Andor Shamrock 303i) with a CCD photo‐detector (Newton CCD detector).

The photoluminescence (PL) measurements were performed at room temperature. The samples were optically excited using a laser beam at a wavelength of 355 nm (neodymium‐doped‐yttrium‐aluminum garnet laser, New Wave Research). The PL emission spectra were collected through a liquid light guide (Newport) connected to a spectrometer (Andor Technology) with a CCD detector and analyzed through computer software. The thermogravimetric analyser (TGA) investigations were performed using a thermogravimetric analyzer (Q 500). For thermogravimetric analysis, ≈5.87 mg (ZnO NWs‐cellulose‐pulp fibers) and ≈4.77 mg (cellulose‐pulp fibers) samples were placed in an aluminum vessel on the pan of the microbalance and were heated from room temperature to 500 °C under constant nitrogen flow with an increase in the temperature at the rate of 20 °C min^−1^.

For the photocurrent measurements, samples with two interdigitated electrodes were optically excited by simulated sunlight from a solar simulator (Oriel Instruments). Before measurements, the solar simulator was calibrated using a reference cell (91150‐KG5 from Oriel Instruments). The photocurrent was measured through a Keithley 2400 source meter. The data were collected and analyzed through a LabVIEW program on a personal computer.

For the sheet resistance measurements, samples with four‐probe electrode configurations were illuminated by simulated sunlight. A circular shadow mask was used to cover the main electrodes and only illuminated only the specific interdigitated electrodes area. The sheet resistance (R_s_) was calculated using the square‐four‐points‐configuration technique as described in ref. [31].

### Photochemical Evolution of Hydrogen Peroxide

The heterogeneous photocatalyst was synthesized by functionalization of cellulose fiber with the metal‐oxide‐based semiconductor ZnO particles. ZnO cellulose pulp paper, deposited on 15 mm × 15 mm glass substrates, was placed on the bottom of a 50 mL borosilicate white beaker, along with 20 mL DI water, and illuminated with a violet source/solar simulator. In the case of the solar simulator, irradiation was from the top with uniform light of the intensity of 0.9 sun (900 W m^−2^) at the point where the sample was placed in a beaker. In the case of violet source (LED, λ_max_ = 400 nm) irradiation, the sample was placed on the light source giving the light intensity of 1200 W m^−2^ at the point where the sample was placed. The irradiation system was equipped with a fan (120 mm diameter) to maintain the room temperature of the water. After every 1 h, 40 µL of the solution was taken to measure H_2_O_2_ concentration with a spectrophotometer.

### Determination of Hydrogen Peroxide Concentration: HRP Assay

The concentration of evolved H_2_O_2_ was measured spectrophotometrically by following the oxidation of 3,3′,5,5′‐Tetramethylbenzidine (TMB) in the presence of horseradish peroxidase (HRP) and citric acid—phosphate buffer solutions. The enzymatic assay was freshly prepared by mixing 993 µL phosphate‐citric acid buffer solution, 5 µL 3,3′,5,5′‐tetramethylbenzidine (TMB), and 2 µL horseradish peroxidase. To detect the concentration of evolved H_2_O_2_ after every 1 h the samples of 50 to 1 µL (depending on hydrogen peroxide concentration) were taken and added to the 250 to 299 µL of freshly prepared enzymatic assay, in every case giving 300 µL of the solution. Then the absorbance of 300 µL solutions was measured at 653 nm with Synergy H1 Microplate reader (BioTek Instruments, Inc.) in 96‐Well Polystyrene flat bottom plates. Then the concentrations were calculated from absorbance using previously determined calibration curve formulas.

## Conflict of Interest

The authors declare no conflict of interest.

## Author Contributions

X.C. and N.H.A. conceived the original idea of the project. X.C. supervised and coordinated all work and finalized the manuscript. N.H.A. performed the synthesis of ZnO nanowires on cellulose pulp fibers, fabricated paper, and the final devices, He also performed SEM, PL, TGA, absorption spectra, stability tests, and photoconductive measurements and analyzed data, and wrote the manuscript. M.B. and I.E. supervised the work on the H_2_O_2_ photosynthesis. N.S. performed experiments for photochemical evolution of hydrogen peroxide and determination of hydrogen peroxide concentration, analyzed the data, and wrote the manuscript. S.S. performed absorption, PL, and photoconductivity measurements. All authors have approved the final version of the manuscript.

## Supporting information

Supporting Information

## Data Availability

The data that support the findings of this study are available from the corresponding author upon reasonable request.
