# Supplementary material for: Toward Photoactive Wallpapers Based on ZnO‐Cellulose Nanocomposites
Source: Glob Chall. 2023 Aug 25;7(9):2300034. doi: 10.1002/gch2.202300034 (PMC10517292; doi:10.1002/gch2.202300034)
Supplement: Supplementary file 1 — Supporting Information [file GCH2-7-2300034-s001.pdf]

Global
Challenges
Open Access

Supporting Information

for *Global Challenges.*, DOI 10.1002/gch2.202300034

Toward Photoactive Wallpapers Based on ZnO-Cellulose Nanocomposites

Naveed Ul Hassan Alvi, Neha Sepat, Samim Sardar, Magnus Berggren, Isak Engquist and Xavier Crispin*

Towards photoactive wallpapers based on ZnO-cellulose nanocomposites

Supplementary Material

The attachment of nanowires with cellulose fibers has solved the problem of leakage of nano/microstructures from the paper. Previously, it was a major challenge to avoid the leakage of the micro- and nanoparticles from the paper over time. This serious problem was limiting their applications to practical devices. We have found that about 38% of the added TPCs and about 19% of added NPs were leaked during the paper fabrication process and about 29% of added TPCs about 33 % of added NPs were leaked after shaking the papers manually 20 times in the air. While ZnO NWs are very stably attached with the cellulose pulp fibers after the papermaking process and also after shaking the paper. Figure S1 (a,b) displays the SEM images of TPCs and NPs that were leaked during the papermaking process and figure S2 (c,d) displays the SEM images of the TPCs and NPs that were leaked after shaking the papers.

Figure S2 displays the SEM images of the ZnO-NWs, TPCs and NPs papers before and after shaking. We shake the fabricated papers manually for 20 times in the air. Figure S2 (a,b) displays the SEM images of ZnO-NWs paper before and after shaking. The SEM images analysis reveals that the NWs are attached to the cellulose pulp fibers very strongly and these are very stable and fully covering the cellulose fibers after shaking the paper. While the TPCs and NPs are unstable and are leaked very easily after shaking the paper as shown in the SEM images in figure S2(c,d) and S2 (e,f), respectively.

The photoconductive responses of the shocked and unshocked ZnO-NWs, TPCs and NPs papers were also measured under irradiance of 1 Sun. ZnO-NWs paper has the same photoresponse before and after shaking while the ZnO-TPCs and ZnO-NPs papers have lost their photoconductivity after shaking as shown in figure S3. The ZnO-NWs paper demonstrates the same and very stable photoresponse after 4 months of time as shown in figure S4. It indicates that the fabricated ZnO-NWs paper has excellent repeatability and durability and have the full potential to be used for long term applications.

Figure S5 shows a stable photocurrent measured at different irradiances (0.01- 1.0 Sun) for NWs paper device while in upper and lower insets are shown for ZnO-TPCs and ZnO-NPs paper devices, respectively. The fabricated devices exhibit different photocurrents under different light irradiances ranging from 0.01–1 Sun. We can explain it by using a formula where total resistance of the device is equal to the sum of the constant resistance (Rc) (generates from contacts/probes/leads) and the variable paper resistance (Rp) (depends on light intensity through the paper). Rp increase with decreasing light irradiance (E), which reveals that the total photocurrent of the device depends on the light irradiance (E),

$$I = \frac{V}{\frac{S}{E} + R_C}$$

where S is the sensitivity of the device, which depends on the design of the device and the light-dependent photoconductivity of the paper. The experimental data (photoconductivity at different light irradiances ranging from 0.01–1 Sun) are fitted well by applying the above equation as shown in figure S5. The Rc of the ZnO NWs device is 0.864 kΩ, while the total light dependent resistance (Rp= S/E) is 16.4 kΩ x Sun. Here, the photocurrent varies approximately linearly with varying light intensities. The total contact and lead resistance of the ZnO-TPCs and ZnO-NPs paper devices are 415 kΩ and 56 MΩ, respectively, while the total light-dependent resistances (Rp= S/E) are 1.5 MΩ x Sun and 38 MΩ x Sun, respectively.

Figure S6: (a,b) displays the SEM images of the ZnO-NWs after photochemical experiments.

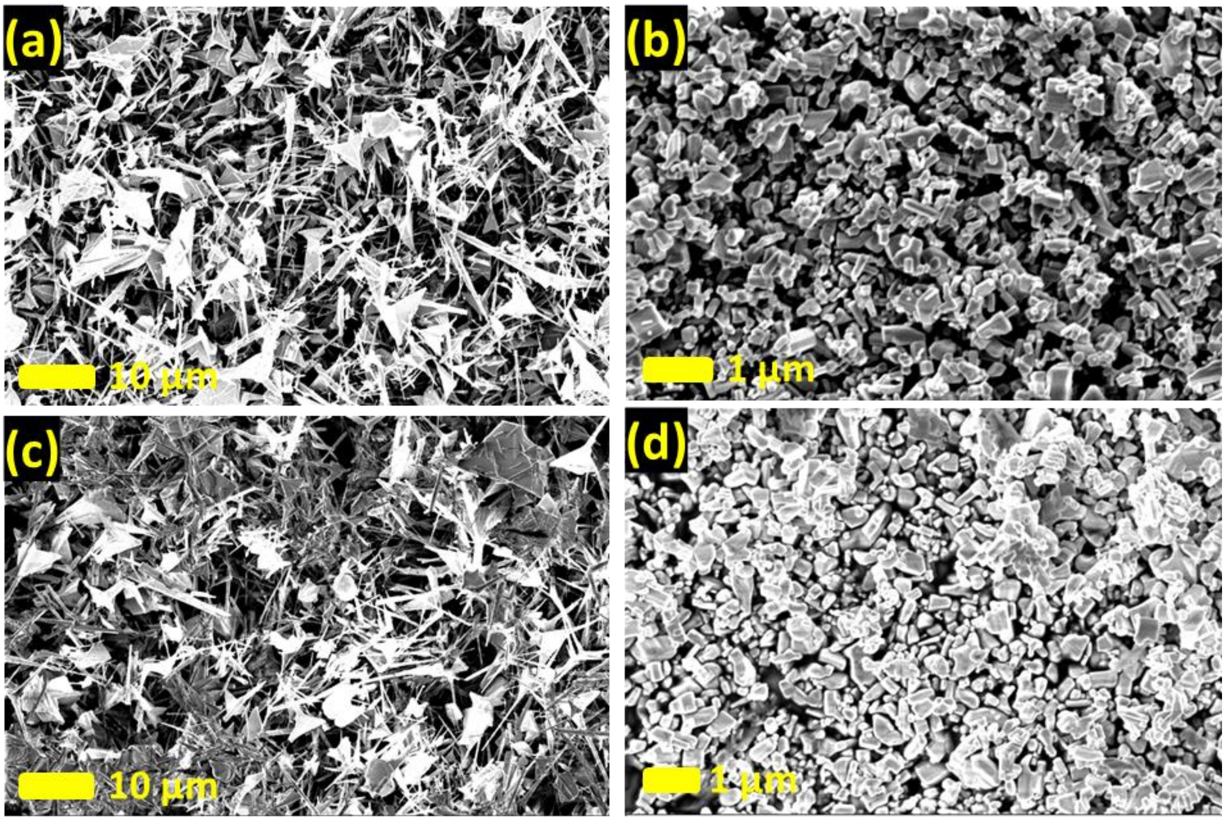

Figure S1: **(a,b)** displays the SEM images of the dropped ZnO-TPCs and ZnO-NPs during the papermaking process and **(c,d)** displays the SEM images of the dropped ZnO-TPCs and ZnO-NPs after shaking the fabricated paper.

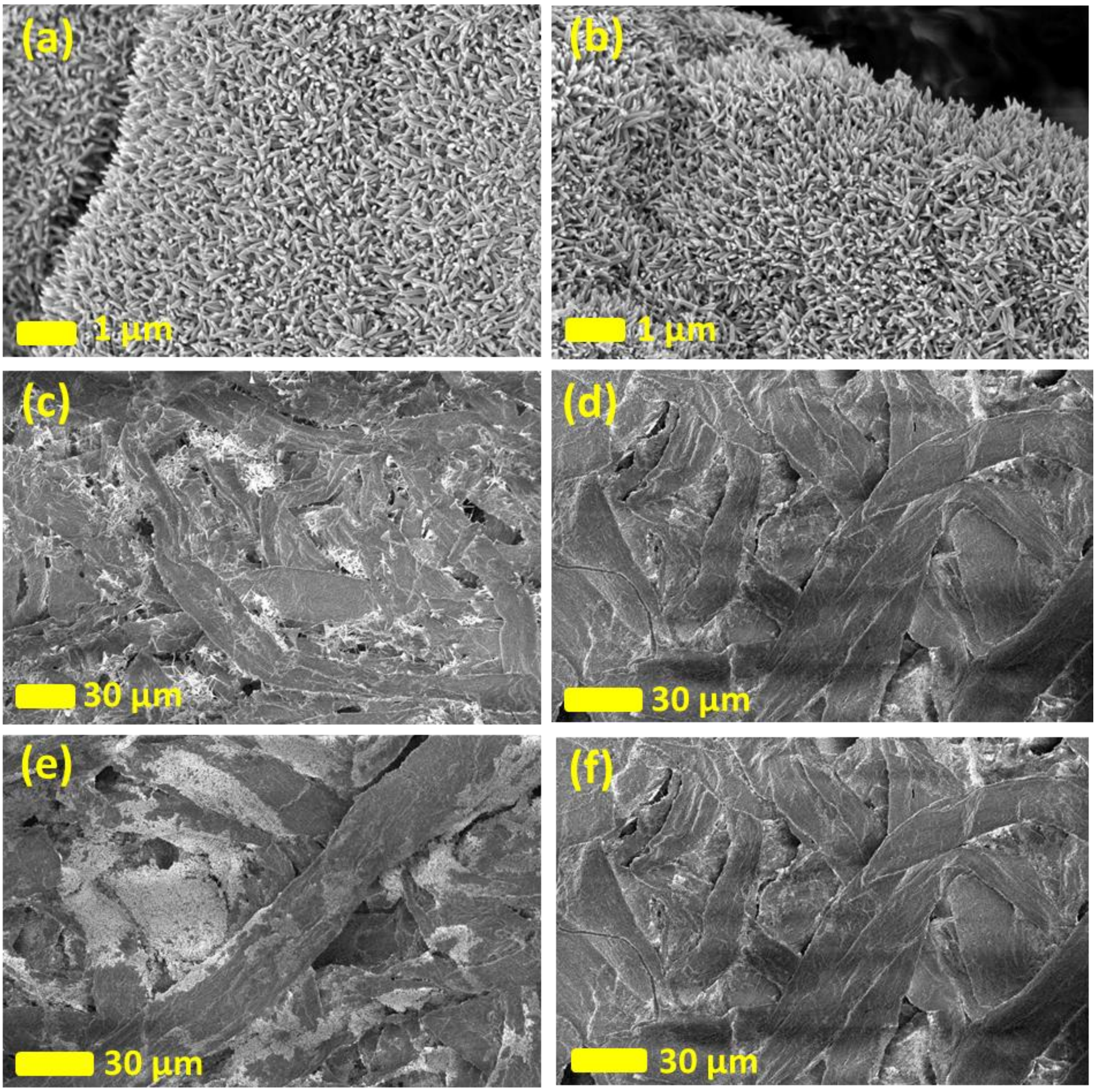

Figure S2: Impact of shaking and stability of ZnO-NWs, ZnO-TPCs and ZnO-NPs papers. **(a,b)** displays the SEM images of the ZnO-NWs paper before and after shaking, similarly, **(c,d)** and **(e,f)** displays the SEM images of ZnO-TPCs and ZnO-NPs papers, respectively.

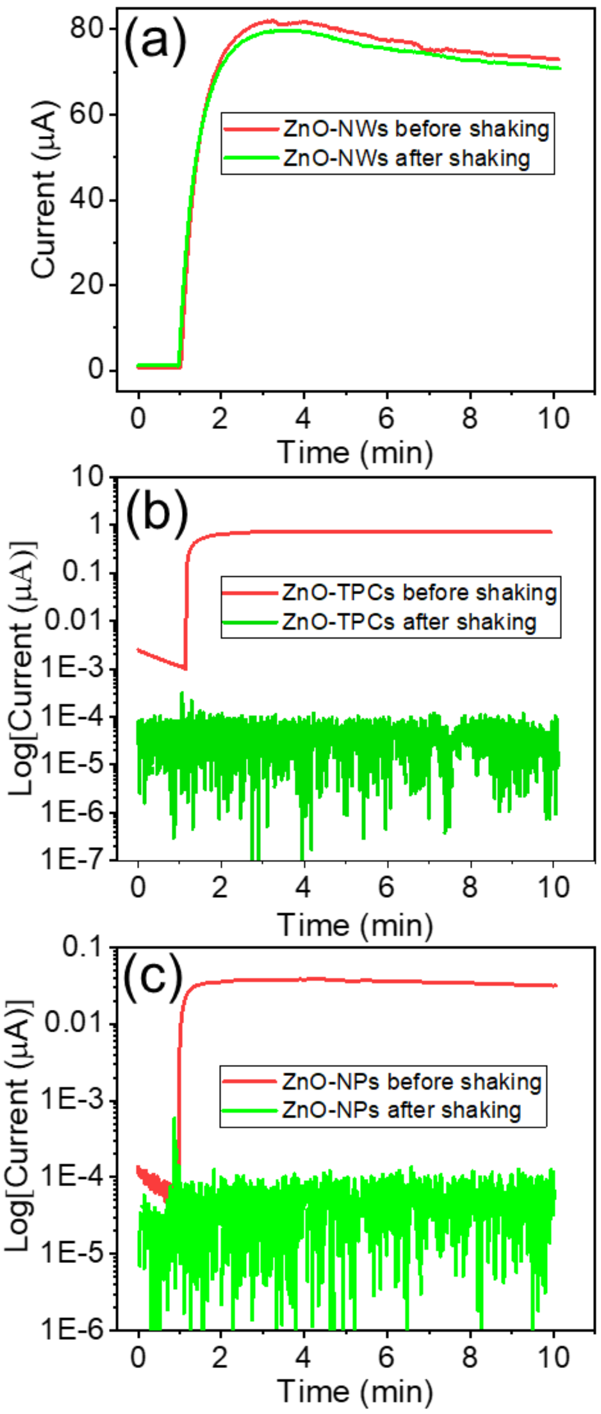

Figure S3: (a) Shows the photocurrent for the ZnO-NWs paper device before and after shaking under an irradiance of 1 Sun at applied potential of 1 V. Similarly, **(b)** and **(c)** shows for the ZnO-TPCs and ZnO-NPs papers devices, respectively .

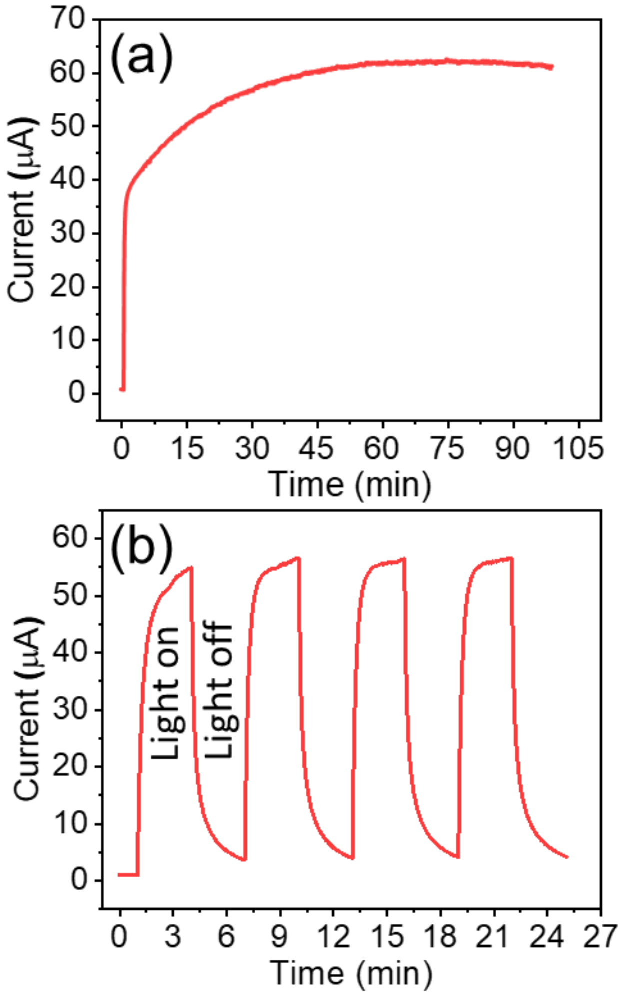

Figure S4: (a,b) Stabilization curve and photo response of the ZnO-NWs, paper device after 4 months at a bias of 1 V and under an irradiance of 1 Sun.

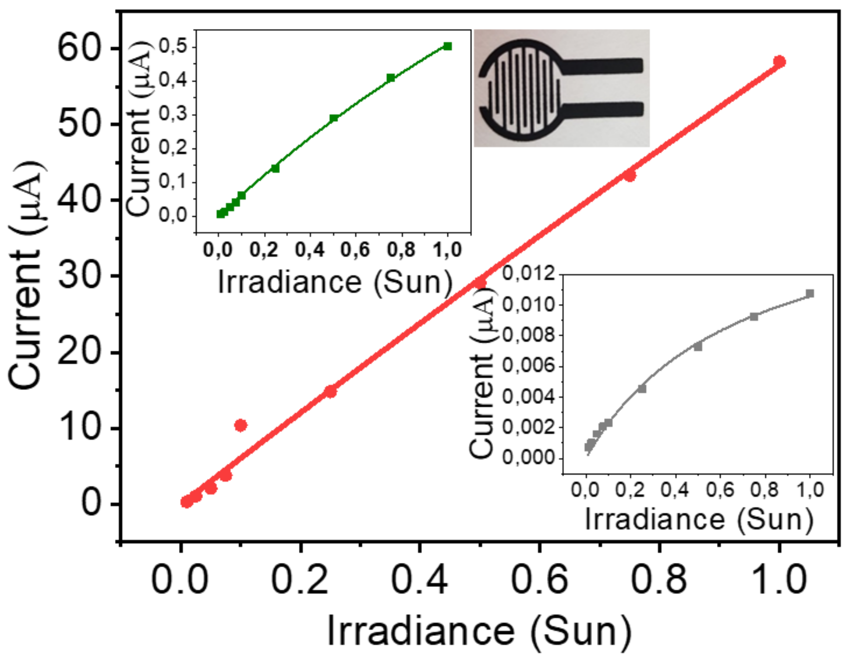

Figure S5: Shows photocurrents versus irradiances (0.01–1 Sun) at 1V for the ZnO-NWs paper device. The data line is a linear fit of the average photocurrents. Similarly, upper inset shows for the ZnO-TPCs paper device and lower inset shows for the ZnO-NPs paper device.

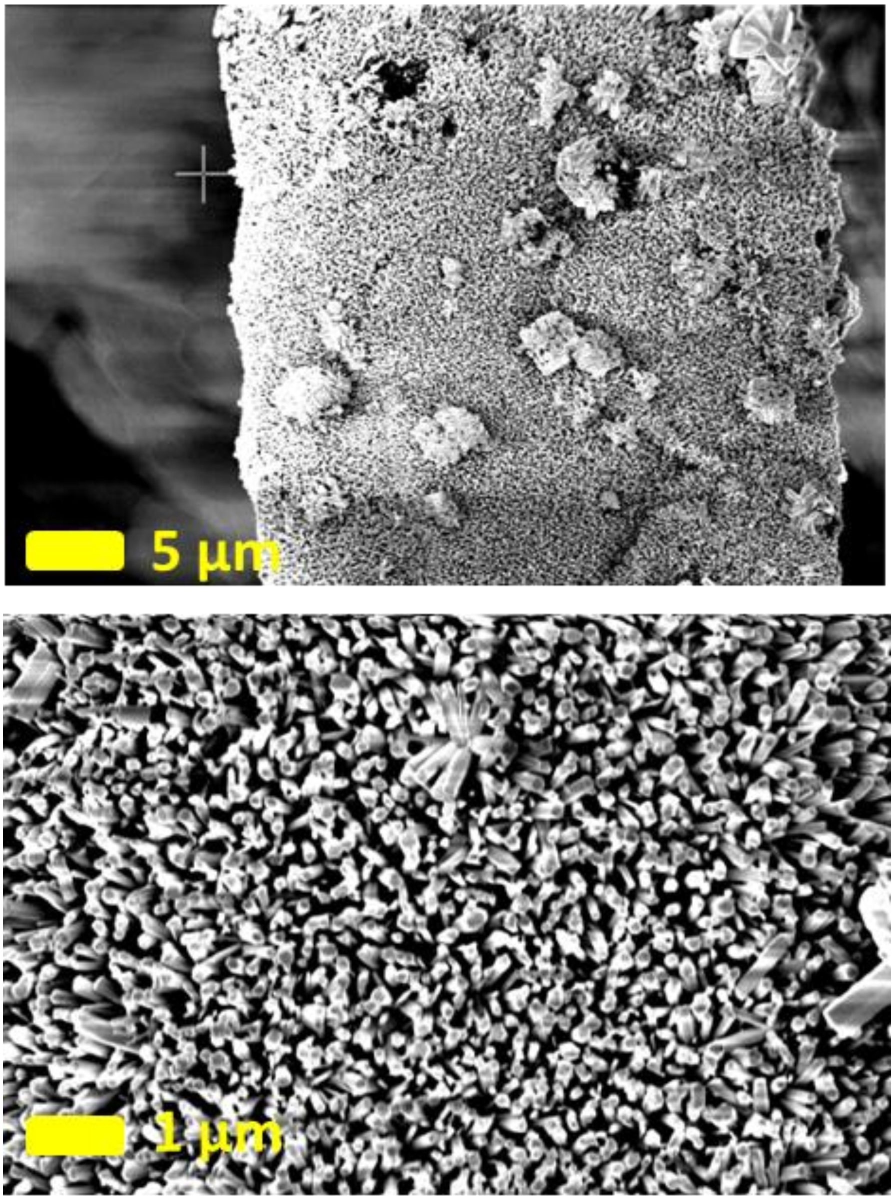

Figure S6: **(a,b)** displays the SEM images of the ZnO-NWs after photochemical experiments.
